# Vildagliptin Attenuates Myocardial Dysfunction and Restores Autophagy via miR-21/SPRY1/ERK in Diabetic Mice Heart

**DOI:** 10.3389/fphar.2021.634365

**Published:** 2021-03-18

**Authors:** Xiaochen Li, Cheng Meng, Fei Han, Juhong Yang, Jingyu Wang, Yanjuan Zhu, Xiao Cui, Minxia Zuo, Jie Xu, Baocheng Chang

**Affiliations:** NHC Key Laboratory of Hormones and Development, Tianjin Key Laboratory of Metabolic Diseases, Chu Hsien-I Memorial Hospital and Tianjin Institute of Endocrinology, Tianjin Medical University, Tianjin, China

**Keywords:** vildagliptin, diabetic cardiomyopathy, microRNA-21, autophagy, SPRY1

## Abstract

**Aim:** Vildagliptin (vild) improves diastolic dysfunction and is associated with a lower relative risk of major adverse cardiovascular events in younger patients. The present study aimed to evaluate whether vild prevents the development of diabetic cardiomyopathy in type 2 diabetic mice and identify its underlying mechanisms.

**Methods:** Type 2 diabetic mouse model was generated using wild-type (WT) (C57BL/6J) and miR-21 knockout mice by treatment with HFD/STZ. Cardiomyocyte-specific miR-21 overexpression was achieved using adeno-associated virus 9. Echocardiography was used to evaluate cardiac function in mice. Morphology, autophagy, and proteins levels in related pathway were analyzed. qRT-PCR was used to detect miR-21. Rat cardiac myoblast cell line (H9c2) cells were transfected with miR-21 mimics and inhibitor to explore the related mechanisms of miR-21 in diabetic cardiomyopathy.

**Results:** Vild restored autophagy and alleviated fibrosis, thereby enhancing cardiac function in DM mice. In addition, miR-21 levels were increased under high glucose conditions. miR-21 knockout DM mice with miR-21 knockout had reduced cardiac hypertrophy and cardiac dysfunction compared to WT DM mice. Overexpression of miR-21 aggravated fibrosis, reduced autophagy, and attenuated the protective effect of vild on cardiac function. In high-glucose-treated H9c2 cells, the downstream effectors of sprouty homolog 1 (SPRY1) including extracellular signal-regulated kinases (ERK) and mammalian target of rapamycin showed significant changes following transfection with miR-21 mimics or inhibitor.

**Conclusion:** The results of our study indicate that vild prevents DCM by restoring autophagy through the miR-21/SPRY1/ERK/mTOR pathway. Therefore, miR-21 is a target in the development of DCM, and vild demonstrates significant potential for clinical application in prevention of DCM.

## Introduction

Diabetes is one of the most common and fastest growing diseases worldwide that is estimated to affect 693 million adults by 2045 (Cho et al., 2018). The prevalence of cardiac dysfunction in individuals with type 1 diabetes mellitus (T1DM) and T2DM has been reported to be as high as 14.5% and 35.0%, respectively, (Konduracka et al., 2013; Bouthoorn et al., 2018). Cardiovascular disease is the leading cause of death in diabetic patients, with approximately 70% mortality due to coronary artery disease. Despite normal coronary state and blood pressure, heart failure occurs in a subset of diabetic patients and is clinically characterized as diabetic cardiomyopathy (DCM) (Poornima et al., 2006). The data from the Framingham study suggest that diabetes is another discrete cause of congestive heart failure and that some form of cardiomyopathy is associated with diabetes (Kannel et al., 1974). Therefore, DCM should be under intensive clinical focus.

The pathogenesis of DCM is complex (Jia et al., 2018). Diabetic myocardial damage caused by hyperglycemia is associated with downregulation of intracellular autophagy (He et al., 2013). Numerous studies have confirmed that miRNAs are involved in the pathophysiological changes of diabetic heart disease, and miRNAs have been identified as key factors in the remodeling of diabetic heart (Guo and Nair, 2017). Accumulating evidence has shown that miR-21 has an inhibitory effect on autophagy (Liu et al., 2015; Huang et al., 2017). In addition, miR-21 is upregulated in diabetic myocardium (Gao et al., 2018; Ghosh and Katare, 2018), but the role of miR-21 in DCM cardiomyocytes has not yet been fully elucidated.

Because cardiovascular (CV) disease is the most common cause of morbidity and mortality in patients with T2DM, the CV safety of any new antidiabetic agent is of critical interest. It was possible to evaluate the cardiovascular and cerebrovascular (CCV) safety of vildagliptin (vild) based on adjudicated CCV events, applying the methodology outlined in the new FDA guidance. The results of meta-analysis of adjudicated events from a large phase III T2DM population showed that vild was not associated with an increased risk of adjudicated CCV events relative to all comparators (placebo or other hypoglycemic agents) in the broad population of type 2 diabetics, including patients at increased risk of CCV events (Schweizer et al., 2010). In addition, a meta-analysis of 17,446 patients showed that the relative risk of prospectively adjudicated major adverse cardiac events (MACEs) in younger (< 65 years) patients with T2DM was significantly reduced by 37% in the vild group compared to the controls (McInnes et al., 2015; Evans et al., 2018). Other studies have shown that vild effectively improves cardiac function in different disease models, such as improving diastolic dysfunction in Dahl salt-sensitive hypertensive rats with HFpEF (Nakajima et al., 2019) and reducing T2DM-induced increase in post-MI acute mortality by restoring the autophagic response (Murase et al., 2015). However, little is known about the mechanisms of the cardioprotective effects of the drug.

Therefore, we hypothesized that vild restores cardiac autophagy by acting on miR-21, thereby protecting cardiac function. In addition, the involvement of the SPRY1/ERK/mTOR pathway in mechanism of action of vild and miR-21 was further explored.

## Materials and Methods

### Animals

Homozygous miR-21 knockout mice (6 weeks-old male mice) that were generated using CRISPR-Cas9 technology (Xue et al., 2019) were kindly provided by Professor Rongxin Zhang’s lab (Basic Medical Research Center, Tianjin Medical University). Selection of homozygous knockout mice by agarose gel electrophoresis (Supplementary Figure S1). Wild-type (WT) mice (C57BL/6J) were obtained from HFK Bioscience Company (Beijing, China).

The experimental protocols were authorized and specifically approved by the Institutional Ethics Committee of the Tianjin Medical University. All experimental mice were maintained on a 12:12 h light–dark cycle at 22 ± 2°C under specific pathogen-free conditions in the animal facility at the Tianjin Medical University (Tianjin, China). The C57/BL6J mice were randomly assigned to five groups: 1) WT normal control group (NC, *n* = 10) were fed a standard diet. 2) WT diabetic group (DM, *n* = 10) were induced by five consecutive daily intraperitoneal injections of streptozotocin (STZ, 30 mg/kg/day, in 0.1 mol/L sodium citrate buffer, pH 4.5; Sigma, MO, United States) after a high-fat diet (60% Kcal high-fat diet, HFD) for 12 weeks. Control mice were injected with an equivalent volume of citrate buffer. Animals with random blood glucose levels ≥16.6 mM were regarded as diabetic as per three independent measurements. 3) After establishing a type 2 diabetic mouse model, WT diabetic mice were treated with vild at a dosage of 15.17 mg/kg/d by oral gavage daily for 10 weeks (DM + vild, *n* = 10). 4) Recombinant adeno-associated virus 9 (rAAV9)-miR-21 was injected into the tail vein of the DM mice, while another group of DM mice was injected with AAV9 NC. The mice were then treated with vild (DM + AAV9 + vild, *n* = 10; DM + AAV9 NC + vild, *n* = 10). The miR-21 knockout mice were randomly assigned to two groups: 1) normal control group (miR-21^−/−^NC, *n* = 10) was fed a standard diet. 2) miR-21^−/−^ diabetic group (miR-21^−/−^DM, *n* = 10): the diabetes model was established as in the WT mice. The animals were sacrificed by cervical dislocation under anesthesia with 5% isoflurane.

### *In vivo* Delivery of rAAV Vectors

The AAV9-miR-21 and AAV9-NC vectors were obtained from Syngen Tech Company (Beijing, China). The vectors were delivered to the myocardium via tail vein injections (total dose of 5 × 10^12^ vg/200 μL per mouse).

### Cardiac Function

A high-resolution echocardiography system (Vevo 770; Visual Sonics, Toronto, ON, Canada) was used to measure the following parameters in anesthetized (1–2% isoflurane) mice after 10 weeks of vild intervention: the left ventricular ejection fraction (LVEF), left ventricle fractional shortening (LVFS), left ventricle internal diameter (LVID), and left ventricular posterior wall thickness (LVPW) were measured during both systole and diastole. The ratio of early (E) to late (A) ventricular filling velocity (E/A ratio) was measured by echocardiography and pulsed Doppler at the level of the mitral valve.

### Cell Culture

Rat cardiac myoblast H9c2 cells (purchased from Beina Chuanglian Biotechnology, Beijing, China) were grown in Dulbecco’s modified Eagle’s medium containing 5.5 mmol/L normal glucose (NG, Gibco, MA, United States) and 10% fetal bovine serum (FBS; Bioind, Israel). The cells were incubated in humidified air (5% CO_2_) at 37°C and treated as follows: 1) Cells in the NG group were incubated in low glucose (5.5 mM) DMEM, 2) Cells in the mannitol (MA) group were incubated in DMEM with 5.5 mM glucose and 27.5 mM MA, 3) Cells in the HG group were incubated in DMEM with 33 mM glucose, and 4) Cells in the HG + vild group were incubated in DMEM with 33 mM glucose and 30 μM vild (MCE, Shanghai, China) for 48 h.

### Western Blot Analysis

Protein isolates were obtained from the cells or cardiac tissues and their concentrations were measured. The protein extracts were then separated using sodium dodecyl sulfate polyacrylamide gel electrophoresis (SDS-PAGE) and transferred to polyvinylidene fluoride membranes (MilliporeSigma, Burlington, MA, United States) followed by overnight incubation at 4°C with primary antibodies against SQSTM1/P62 (1:1,000; CST), LC3 (1:1,000; Proteintech), SPRY1 (1:1,000; Bioss), connexin-43 (Cx43; 1:1,000; CST), *p*-ERK (1:1,000; CST), t-ERK (1:1,000; CST), *p*-mTOR (1:1,000; CST), t-mTOR (1:1,000; CST), *β*-tubulin (1:1,000; Abclonal), and GAPDH (1:1,000; Abclonal). Then the membranes were reacted with secondary antibodies. Chemiluminescence signals were recognized by ECL reagents (Advansta, CA, United States). Blots were quantified with ImageJ software.

### Immunohistochemical Staining

Formalin-fixed cardiac samples were embedded in paraffin blocks. Representative sections (5 µm thick) were incubated overnight with the primary antibodies against SQSTM1/P62 (1:200), LC3 (1:200), and Cx43 (1:100; CST). Slides were developed with diaminobenzidine (DAB) kits after incubation with the appropriate secondary antibodies.

### Immunofluorescence Staining

Cells were permeabilized with 1% Triton X-100 and fixed with paraformaldehyde. The cells were then blocked with 5% BSA, followed by incubation with primary antibodies against SQSTM1/P62 (1:50; CST, MA, United States) at 4°C overnight. The cells were incubated with Alexa Fluor-488-conjugated secondary antibodies for 1 h at room temperature. Nuclei were stained with DAPI reagents. Fluorescence signals were detected using confocal fluorescence microscopy (Leica Microsystems, Germany).

### Morphometric Measurements

Hematoxylin and eosin (HE) staining was used for the general assessment of cardiac morphology. Masson staining was used to detect interstitial fibrosis. Images were visualized using a light microscope and a digital imaging system.

### RNA Extraction and Quantification of Gene Expression

Total RNA was extracted from the treated cells with E. Z.N.A.^®^ Total RNA Kits (Omega, GA, United States). cDNA was synthesized using a reverse transcription system kit (Thermo, United States). Real-time qPCR was performed using SYBR Green PCR kits (Sangon Biotech, Shanghai). The primers used are listed in Supplementary Table S4.

### Transfection of miRNA Inhibitor and Mimics

MiR-21 inhibitor, mimics, and the homologous negative control were obtained from GenePharma (Shanghai, China). Cells were transfected using Lipofectamine 2000 reagent (Invitrogen, CA, United States) at a final concentration of 50–100 mM according to the manufacturer’s instructions.

### Statistical Analysis

Data are expressed as the mean ± standard error of the mean (SEM). The analysis was performed using SPSS 22.0 software. Statistical differences between two groups were evaluated using unpaired Student’s *t*-test. Comparisons between multiple groups were analyzed by one-way ANOVA, followed by the LSD *post hoc* test. Statistical significance was determined at *p* < 0.05.

## Results

### Effects of Vild Treatment on Cardiac Pathological and Functional Changes in Diabetic Mice

**FIGURE 1:**
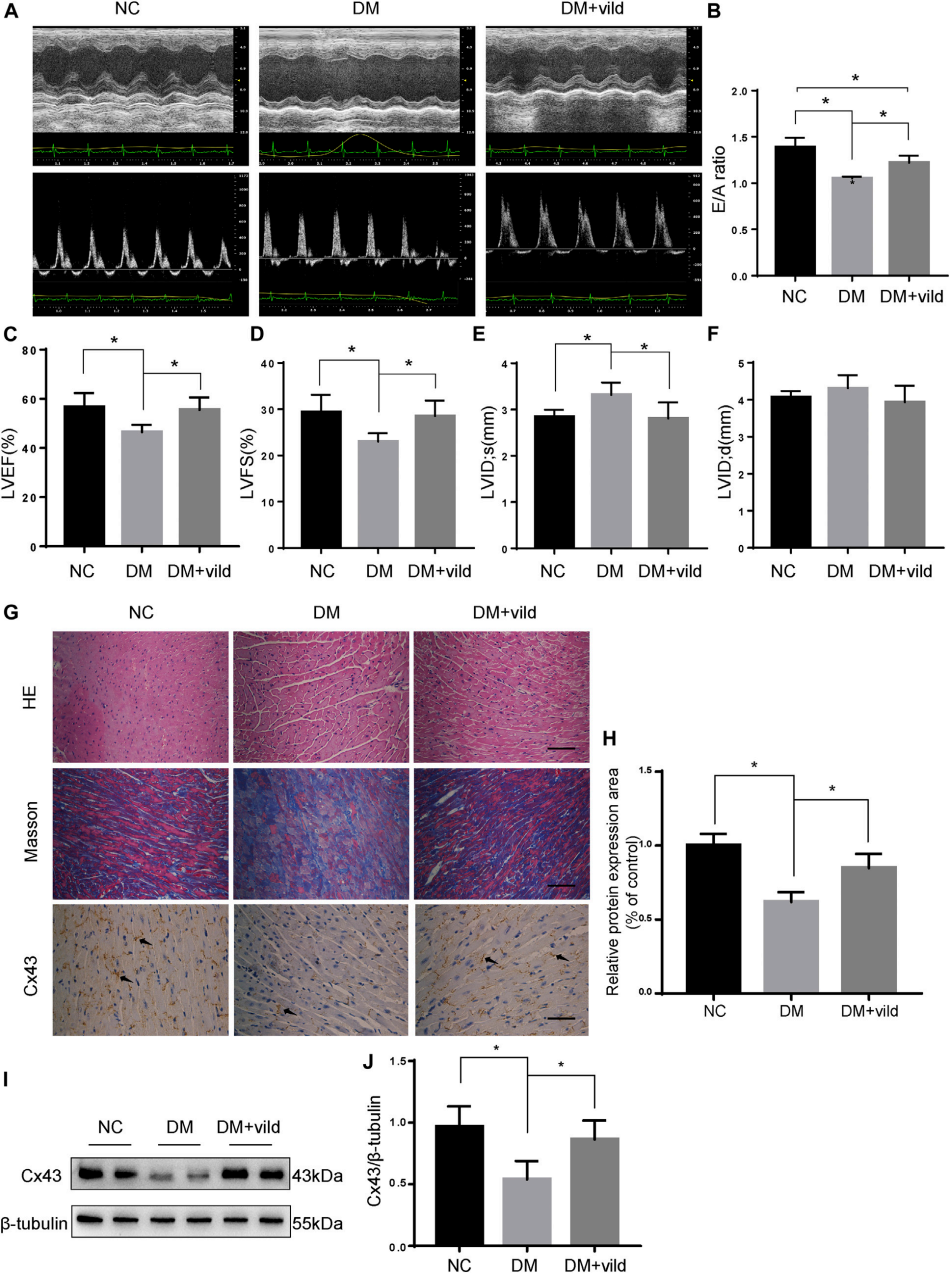
Vild protects heart function and alleviates hyperglycemia-induced pathological changes. **(A)** M-mode echocardiograms and pulse-wave Doppler echocardiograms were used to examine cardiac function. Quantitative analysis of E/A **(B)**, LVEF **(C)**, LVFS **(D)**, LVIDs **(E)**, and LVIDd **(F)**. Data are presented as mean ± standard deviation (SD). **(G)** Representative images of cardiac sections stained with HE, Masson, and immunohistochemically stained for Cx43. Scale bars in the right lower corner represent 20 μm. **(H)** Immunochemistry analysis of Cx43. **(I)** Representative western blotting (WB) images of Cx43. **(J)** Quantification of Cx43 protein levels. NC, normal control; DM, diabetic group; DM + vild, diabetic mice with vild administration; Cx43, connexin 43. Data are reported as mean ± standard error of mean (SEM). *p < 0.05.

Echocardiography was performed to assess cardiac function at the end of the experiment (Figures 1A–F). Compared to the NC group, the E/A value, LVEF, and FS were significantly decreased in the DM group. The LVIDs of the DM group were significantly higher than those of the NC group. Compared to the DM group, the E/A value, LVEF, and FS were significantly increased in the vild treatment group. Treatment with vild also simultaneously inhibited the increase in LVID.

HE and Masson staining were performed to examine the changes in cardiac pathology (Figure 1G). Light microscopic images revealed hypertrophied cardiomyocytes and increased extracellular matrix in the DM group. Masson’s trichrome staining revealed increased interstitial fibrotic areas in the DM group compared to the NC group, whereas vild treatment alleviated cardiac fibrosis in the DM mice.

Cx43 is the predominant connexin expressed by cardiomyocytes and is crucial for cell-cell communication and cardiac function (Severs et al., 2004). Results of IHC and WB analysis showed that the expression of Cx43 was reduced in the DM group compared to that in the NC group. However, the expression of Cx43 was increased in the DM + vild group (Figures 1G–J).

### Effects of Vild on Autophagy *in vivo* and *in vitro*

**FIGURE 2:**
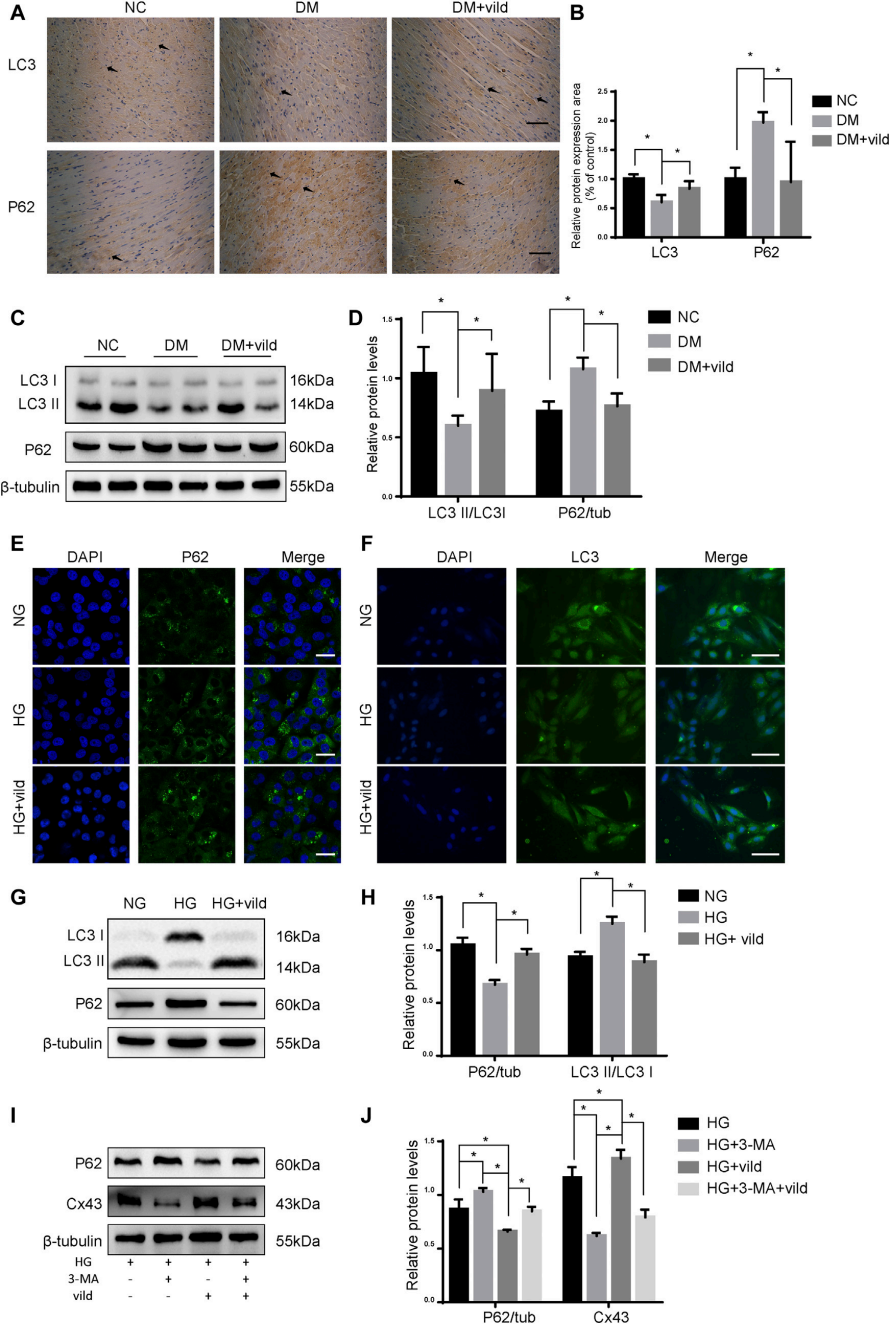
Vild treatment restores autophagy *in vivo* and *in vitro*. **(A)** Immunohistochemical staining of P62 and LC3 in cardiac sections. Scale bars in the right lower corner represent 20 μm. **(B)** Immunochemistry analysis of LC3 and P62. **(C)** Representative WB images of P62 and LC3. **(D)** Quantification of P62 and LC3 protein levels. **(E)** Immunofluorescence staining of P62 in H9c2 cells. Scale bars in the right lower corner represent 5 μm. **(F)** Immunofluorescence staining of P62 and LC3 in HMCs. Scale bars in the right lower corner represent 50 mm **(G)** Representative WB images of P62 and LC3 in H9c2 cells. **(H)** Quantification of P62 and LC3 protein levels. *p < 0.05. **(I)** Representative western blotting images of P62 and Cx43. **(J)** Quantification of P62 and Cx43 protein levels.

As shown in Figures 2A,B, IHC and WB analysis revealed that P62 level was notably decreased in the DM + vild group. The aggregation of LC3 was significantly elevated in the DM + vild group compared to the DM group. However, WB analysis revealed that vild rescued LC3 aggregation and restored the ratio of LC3 II to LC3 I in the DM + vild group (Figures 2C,D). Furthermore, vild reversed the reduction in autophagy in DM mice. We also confirmed the effect of vild on autophagy in the rat cardiac myoblast cell line, H9c2. As shown in Figure 2E, P62 levels were significantly increased in the HG group, but were restored in HG + vild group. Punctate LC3 expression was decreased in the HG group, but increased in HG + vild group (Figure 2F). We also validated the results with WB (Figures 2G,H). Results of the WB analysis (protein level) were the identical to that obtained from IF analysis.

As shown in Figures 2I,J, compared to the HG group, the levels of P62 were significantly upregulated and Cx43 was significantly downregulated in the HG + 3-MA group. In addition, we compared the protein levels in HG + vild group with those in HG + vild + 3-MA group and found that the levels of P62 were increased, whereas that of Cx43 was decreased following 3-MA treatment, indicating that the cardioprotective effects of vild were blocked by 3-MA and were autophagy related.

### miR-21 is Upregulated in DCM

**FIGURE 3:**
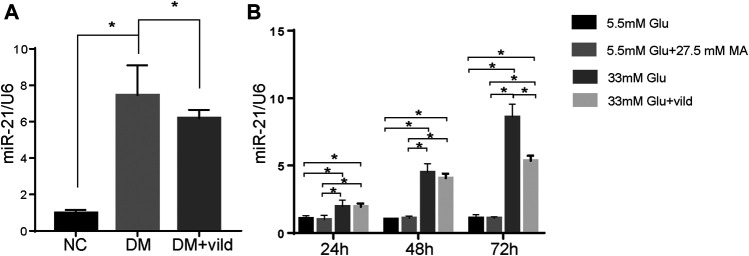
miR-21 is upregulated in diabetic cardiomyopathy. **(A)** Expression of miR-21 of each group in tissues. **(B)** Expression of miR-21 in different groups of H9c2 cells. miR-21 expression level is expressed relative to that of U6 snRNA. Error bars represent SEM. *p < 0.05.

We hypothesized that vild restores autophagy via miRNA. Therefore, we quantified miR-21 in NC, DM, and DM + vild groups. miR-21was significantly increased in DM but reduced in DM + vild group (Figure 3A). We then semi-quantified miR-21 in cells treated with NG, MA, HG, and HG + vild, respectively. We found that there were significant differences between NG and HG, but no obvious changes in miR-21 levels between NG and MA. Compared to the HG group, miR-21 showed a significant decrease in HG + vild group (Figure 3B).

### miR-21 is Involved in DCM by Regulating Autophagy

**FIGURE 4:**
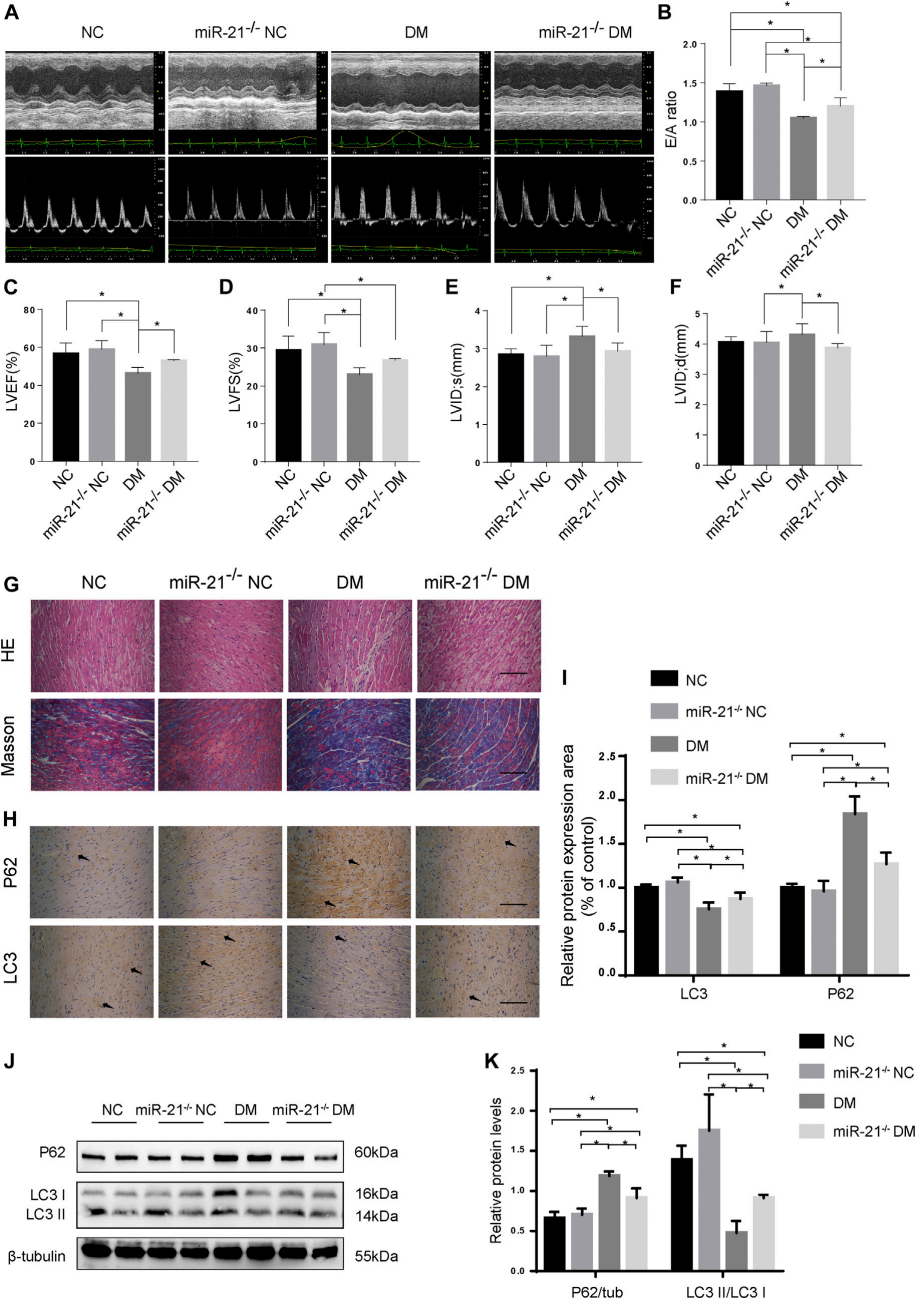
miR-21 regulates autophagy in DM mice. **(A)** M-mode echocardiograms and pulse-wave Doppler echocardiograms was used to examine cardiac function. Quantitative analysis of E/A **(B)**, LVEF **(C)**, LVFS **(D)**, LVIDs **(E)**, and LVIDd **(F)**. Data were presented as mean ± SD. **(G)** Representative images of HE and Masson-stained cardiac sections. Scale bars in the right lower corner represent 20 μm. **(H)** Immunohistochemical staining of P62 and LC3 in cardiac sections. Scale bars in the right lower corner represent 20 μm. **(I)** Immunochemistry analysis of LC3 and P62. **(J)** Representative western blotting images of P62 and LC3 in cardiac tissues. **(K)** Quantification of P62 and LC3 protein levels. *p < 0.05.

To investigate whether miR-21 is involved in DCM pathogenesis, we established a diabetic model using miR-21 knockout mice, similar to WT mice, and performed echocardiography at the end of the experiment (Figures 4A–F). The results showed no significant difference in cardiac function between the miR-21^−/−^ NC and WT NC groups. The EF and E/A values of the miR-21^−/−^DM group were significantly higher than those of the WT DM group. LVIDs and LVIDd in the miR-21^−/−^DM group were lower than those in the WT DM group. Thus, these results indicate that knocking out miR-21 improves cardiac systolic and diastolic functions and reduces left ventricular hypertrophy.

Myocardial HE and Masson’s trichrome staining (Figures 4G,H) showed that compared to the WT DM group, myocardial hypertrophy and fibrosis were significantly reduced in the miR-21^−/−^DM group. Moreover, we found decreased levels of P62 and increased accumulation of LC3 in the miR-21^−/−^ DM group compared to the DM group. In contrast, miR-21 KO decreased P62 expression and the ratio of LC3 II to LC3 I was restored (Figures 4J,K). These results support the hypothesis that miR-21 downregulates autophagy in the diabetic myocardium.

### miR-21 Downregulates Autophagy via the SPRY1/ERK/mTOR Pathway

**FIGURE 5:**
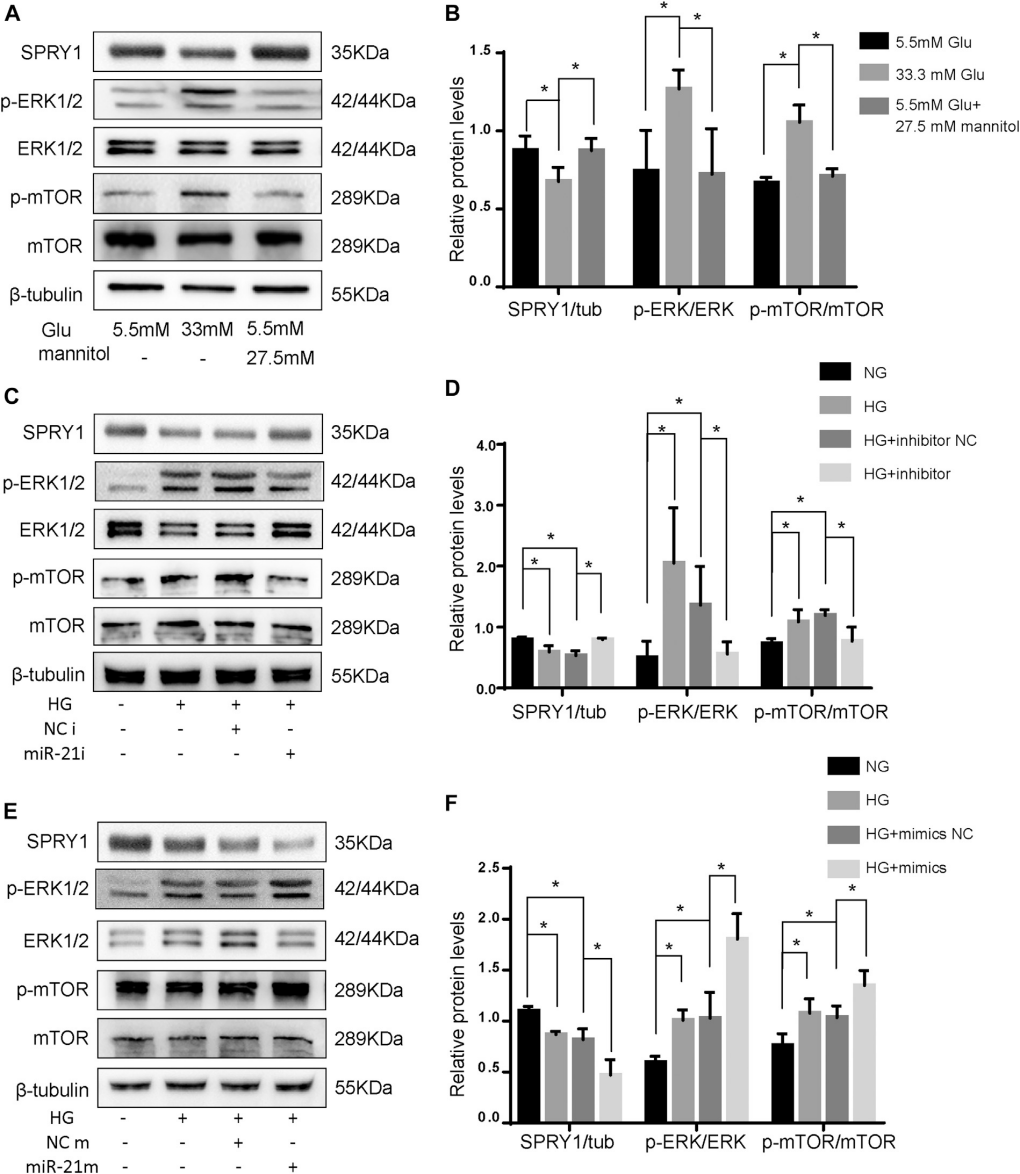
miR-21 regulates autophagy via SPRY1/ERK/mTOR pathway under HG. **(A)** Representative WB images of SPRY1, *p*-ERK, ERK, *p*-mTOR, and mTOR in H9c2 cells **(B)** Quantification of SPRY1, *p*-ERK, ERK, *p*-mTOR, and mTOR protein levels. *p < 0.05. **(C, E)** Representative WB images of SPRY1, *p*-ERK, ERK, *p*-mTOR, and mTOR in H9c2 cells. **(D, F)** Quantification of SPRY1, *p*-ERK, ERK, *p*-mTOR, and mTOR protein levels. *p < 0.05. NG, normal glucose; HG, high glucose (33 mM glucose); miR-21i, miR-21 inhibitor; miR-21m, miR-21 mimics; NCi, miR-21 inhibitor negative control; NCm, miR-21 mimic negative control.

To confirm the differentially expressed pathways in H9c2 cells following HG treatment, the levels of the SPRY1/ERK/mTOR signaling pathway members were examined by WB. The results showed that compared to the NG group, the expression of SPRY1 was significantly decreased in the HG group, whereas the expression of *p*-ERK and *p*-mTOR were increased. However, the MA control group did not show any apparent difference in H9c2 cells relative to the NG group (Figures 5A,B). These results suggest that the SPRY1/ERK/mTOR pathway was inhibited in H9c2 cells following HG treatment.

To further clarify whether miR-21 regulates the differentially expressed pathway, we transfected H9c2 cells with miR-21 mimics (miR-21 m) and miR-21 inhibitor (miR-21i). The protein levels of SPRY1 were significantly increased after miR-21i transfection, and those of *p*-ERK and *p*-mTOR were significantly decreased (Figures 5C,D). On the contrary, the result of miR-21 m transfection was opposite to that of mir-21i transfection (Figures 5E,F). These results indicate that miR-21 regulates autophagy through the SPRY1/ERK/mTOR pathway.

### Vild Restored Autophagy and Prevented Diabetic Cardiomyopathy by Regulating the miR-21/SPRY1/ERK/mTOR Pathway

**FIGURE 6:**
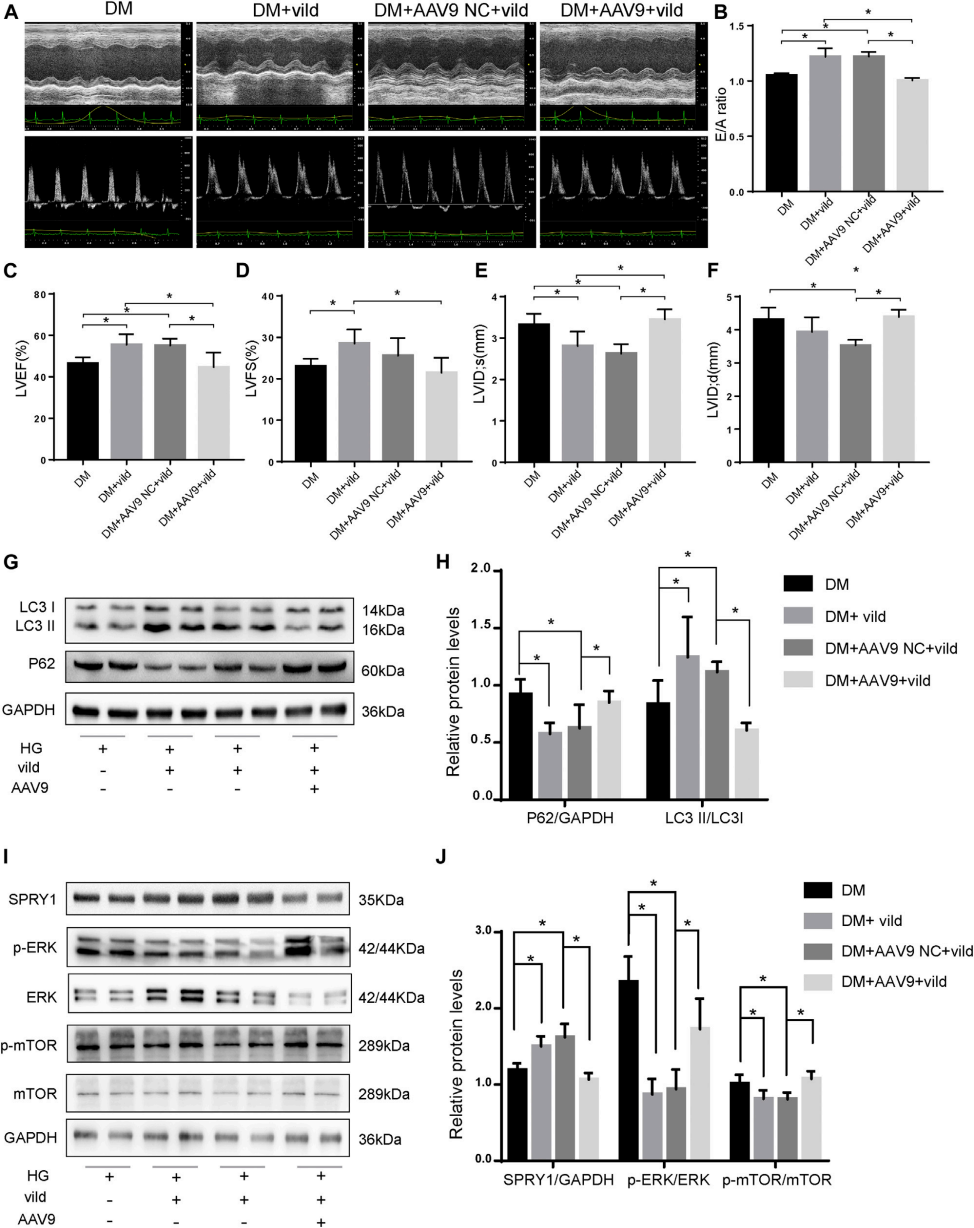
Vild prevents DCM and autophagy by regulating the miR-21/SPRY1/ERK/mTOR pathway. **(A)** M-mode echocardiograms and pulse-wave Doppler echocardiograms were used to examine cardiac function. Quantitative analysis of E/A **(B)**, LVEF **(C)**, LVFS **(D)**, LVIDs **(E)**, and LVIDd **(F)**. Data are presented as mean ± SD. **(G)** Representative WB images of P62 and LC3. **(H)** Quantification P62 and LC3 protein levels. **(I)** Representative WB images of SPRY1, *p*-ERK, ERK, *p*-mTOR, and mTOR of cardiac tissues. **(J)** Quantification of SPRY1, *p*-ERK, ERK, *p*-mTOR, and mTOR protein levels. *p < 0.05. DM + AAV9 NC + vild, diabetic mice with no-load AAV9 and vild; DM + AAV9 + Vild, diabetic mice with AAV9-miR-21 and vild.

We generated miR-21 overexpressing mice by injecting the mice with AAV9-miR-21. The expression of miR-21 increased after 4 weeks (Supplementary Figure S2). The results of echocardiography (Figures 6A–F) revealed that the DM + AAV9 + vild group had lower E/A values, LVEF, and LVFS compared to the DM + vild group, indicating a decrease in diastolic and systolic functions. In addition, the LVID value increased in the DM + AAV 9 + vild group compared to that in the DM + vild group, indicating that the wall thickening was aggravated. Thus, miR-21 overexpression counteracted the positive regulation of cardiac function.

After transfection of the miR-21 overexpressing virus, the expression of P62 was significantly higher in the DM + AAV9 + vild group compared to that in the DM + vild group. The expression of LC3II/LC3I was significantly lower than that in the DM + vild group, indicating that the level of autophagy was decreased (Figures 6G,H). This indicates that, following overexpression of miR-21, the effect of vild on the recovery of autophagy in diabetic mice was abrogated. Furthermore, the expression of SPRY1 in the DM + AAV9 + vild group was significantly lower compared to that in the DM + vild group. The expression of *p*-ERK and *p*-mTOR was higher in the DM + AAV9 + vild group than that in the DM + vild group (Figures 6I,J).

## Discussion

In this study, we provide *in vivo* evidence that miR-21 is a vital factor for DCM in mice challenged with STZ/HFD. Importantly, miR-21 expression was elevated in the myocardial tissues of diabetic mice. In addition, cardiac functions were significantly improved in the miR-21-knockout diabetic mice compared to that in C57 diabetic mice. We found that vild activates autophagy in diabetic mice by inhibiting the expression of miR-21 and activation of the SPRY1/ERK/mTOR pathway, thereby preventing DCM.

Vildagliptin, a dipeptidyl peptidase 4 inhibitor, is an oral hypoglycemic drug that reduces hyperglycemia in T2DM (Mari et al., 2005). The safety and efficacy of vild as monotherapy or in combination with other oral antidiabetic or basal insulin has been well established. In addition to their hypoglycemic effects, the cardiovascular benefits of hypoglycemic drugs are under intensive scrutiny currently. Vild improves insulin resistance in rats and endothelial function in patients with T2DM (van Poppel et al., 2011; Apaijai et al., 2014). Vild has also been shown to play a beneficial role in the heart, in various disease models. Further, it preserves left ventricular function and reduces infarct size of cardiac I/R injury by preventing mitochondrial dysfunction (Chinda et al., 2013; Chinda et al., 2014). Moreover, vild reduced myocyte hypertrophy and perivascular and cardiac fibrosis in isoproterenol-treated rats (Miyoshi et al., 2014). In rats with insulin resistance induced by a high-fat diet, vild prevented cardiac mitochondrial membrane depolarization and attenuated cardiac dysfunction (Apaijai et al., 2012; Apaijai et al., 2013). However, no study has evaluated the effect of vild on cardiac function in a model of T2DM. In our study, the E/A value and the ejection fraction were decreased in the DM group at the end of the 22nd week, indicating diastolic dysfunction and impaired myocardial contractile function. This phenomenon matched that of the late phase of DCM, indicating the successful establishment of our model. Furthermore, the E/A and ejection fraction values in the DM + vild group were significantly higher compared to those in the DM group, indicating a significant protective effect of vild on cardiac function.

A recent study reported that vild improved survival after myocardial infarction (MI) in Otsuka Long-Evans Tokushima fatty (OLETF) rats by restoration of the autophagic response (Murase et al., 2015), which was consistent with our results. In our study, autophagy was decreased in the heart of DM mice and in H9c2 cells treated with high glucose. Autophagy is a homeostatic mechanism that maintains cardiac structure and function (Martinet et al., 2007). Autophagy may be protective or detrimental depending on the cell type, cellular environment, and the extent of autophagy. Several studies have shown that the activation of autophagy by systemic administration of drugs [metformin (He et al., 2013), resveratrol (Kanamori et al., 2015), and fenofibrate (Zhang et al., 2016)] is beneficial in improving diabetes-induced cardiac dysfunction. To further explore whether inhibition of autophagy mitigates vild-induced cardioprotective effects, we applied 3-MA in the HG + vild group. We compared the protein levels in HG + vild with those in HG + vild + 3-MA and found that the level of P62 was increased whereas that of Cx43 was decreased following 3-MA treatment, indicating that the restoration of cx43 expression by vild was blocked by 3-MA indicating that the vild-mediated effect was autophagy related.

However, this study also revealed that vild did not act on autophagy directly, but rather through the intermediary, miR-21. miR-21 plays different roles in different diseases. In vascular disease, miR-21 knockout exacerbated AngII-induced TAAD formation in mice through the TGF-β pathway (Huang et al., 2018). Several studies have shown that miR-21 expression is upregulated in the ventricles under diabetic conditions (Guo and Nair, 2017; Lopes et al., 2017). In this study, we found that the expression level of miR-21 was upregulated in the heart tissue of diabetic mice and in H9c2 cells treated with high glucose, which was consistent with previous studies (Thum et al., 2008). miR-21 plays a key role in myocardial fibrosis. Liu et al. demonstrated that the expression of miR-21 was significantly increased in fibroblasts, leading to increased collagen synthesis and phosphorylation of p38 MAPK (Liu et al., 2014), thereby suggesting a key role of miR-21 in DCM. To confirm the role of miR-21 in DCM, miR-21-knockout mice were used to establish a diabetic model (miR-21^−/−^DM) similar to WT (WT DM) mice. The results indicated that contraction and diastolic functions were protected whereas wall hypertrophy was alleviated in the miR-21 knockout mice. A previous study reported that miR-21 prevents early DCM by attenuating impairments in diastolic dysfunction and cardiac hypertrophy, by reducing the production of ROS via gelsolin (Dai et al., 2018). In this study, we established an advanced DCM model rather than an early-stage DCM model. Various DCM stages and the different interventions may have different effects on H9c2 cells and this poses a major challenge in interpretation of the results. Furthermore, blood glucose levels in the miR-21^−/−^DM mice were lower than those of WT DM mice, which may be related to an increase in insulin sensitivity and the regulation of various key metabolic processes involved in fatty acid uptake, gluconeogenesis, and glucose output by miR-21 knockout (Calo et al., 2016). However, when the DM mice were injected with heart-specific AAV9 carrying miR-21, no significant change was detected in FBG in the DM + AAV 9 + vild group as compared to the DM + vild group, whereas the E/A ratio and LVEF was decreased and the ventricular wall hypertrophy was aggravated. Thus, miR-21 appears to have a negative effect on cardiac function via mechanisms that do not involve the blood glucose factors. Generally, knockout mice were affected by confounding factors. As mentioned above, the lack of miR-21 in the liver caused the difference in blood glucose level. In order to study the specific role of miR-21 in the heart, we will take the heart-specific knockout mice as an experimental model in the follow-up experiments.

Using the AAV9 gene delivery model, we provided critical genetic validation. The results showed that the level of autophagy in DM mice treated with vild after overexpression of miR-21 was significantly lower than that in the mice treated with vild alone, and the expression of SPRY1 was also significantly decreased, while *p*-ERK and *p*-mTOR were significantly elevated. This data suggests that vild may activate the expression of the SPRY1/ERK/mTOR pathway by downregulating miR-21 level, thereby increasing autophagy levels and improving cardiac function. Consistent with our results, miR-21 has been reported to activate *p*-ERK (Huang et al., 2018) that is effectively decreased by vild treatment (Apaijai et al., 2016), which is consistent with our results. Cells were transfected with SPRY1-siRNA to confirm the function of SPRY1 (Supplementary Figure S3). The results indicated that vild regulated autophagy through the SPRY1/ERK/mTOR pathway.

DCM has multiple pathophysiologic triggers. Hyperglycemia is one of the most important causes of diabetes-related cardiovascular disease that often leads to the development of cardiac dysfunction (Ren et al., 1999). In our *in vitro* studies, we used high glucose (33 mM) intervention in H9c2 cells and found that mir-21 expression was increased whereas the level of autophagy was decreased, which was consistent with the results of our animal experiments. Therefore, we focused on the effects of glucose in further studies. Many studies have focused on the effects of palmitate in DCM (Wen et al., 2013; Pang et al., 2015). However, studies have found that the confounding effects of the classical risk factors that coexist in diabetes, such as hyperglycemia, insulin resistance, hyperlipidemia, metabolic disturbances, and neurohormonal activation, combined with other risk factors, may promote DCM progression. PA-treated H9c2 cells presented with enhanced levels of ER stress, apoptosis, and lipid deposition, as well as impaired autophagy (Wu et al., 2020). Therefore, to better simulate T2DM, we will maintain the myocytes in “diabetic like” medium of insulin-high glucose (Davidoff and Ren, 1997) or PA-high glucose (Hu et al., 2019) to study the pathological mechanisms in further studies.

## Data Availability

The original contributions presented in the study are included in the article/Supplementary Material, further inquiries can be directed to the corresponding authors.

## Supplementary Material

FIGURE S1: Selection of homozygous knockout mice by agarose gel electrophoresis. Mice with a band at 713 bp were identified as homozygous miR-21-knockout mice; mice with a band at 782 bp were identified as WT mice; double band indicated heterozygous mice.

FIGURE S2: Transfection efficiency of adeno-associated virus 9 (AAV9) vector. EGFP expression in frozen sections of the heart was observed under a microscope.

FIGURE S3: Vild regulated autophagy by SPRY1. **(A)** Representative WB images of P62 and Cx43 for different groups after transfection with SPRY1-siRNA. **(B)** Quantification P62 and Cx43 protein levels.

FIGURE S4: Random blood glucose and OGTT of each group. **(A)** Random blood glucose in each group at 12 weeks and 22 weeks. **(B)** OGTT results in mice at 22 weeks. **(C)** AUCg values determined by OGTT. Data are reported as mean ± SEM. a p < 0.05 vs. NC group; b p < 0.05 vs; c p < 0.05 vs. DM + vild group; d p < 0.05 vs.DM+AAV9 NC + vild group; e p < 0.05 vs.DM+AAV9 + vild group; f p < 0.05 vs.miR-21^−/−^ NC group.
