# Supplementary material for: Vildagliptin Attenuates Myocardial Dysfunction and Restores Autophagy via miR-21/SPRY1/ERK in Diabetic Mice Heart
Source: Front Pharmacol. 2021 Mar 18;12:634365. doi: 10.3389/fphar.2021.634365 (PMC8013777; doi:10.3389/fphar.2021.634365)
Supplement: Supplementary file 1 [file table1.docx]

**Table S1. Physical and biochemical parameters of mice in NC, DM and DM+vild group (mean ± SD).**

|  | NC | DM | DM+vild |
| --- | --- | --- | --- |
| Body weight（g） | 33.12±6.68 | 34.82±1.28 | 32.44±5.38 |
| FBG（mmol/L） | 5.68±1.41 | 18.47±3.22* | 9.13±2.15*^#^ |
| TG（mmol/L） | 1.41±0.20 | 4.64±0.96* | 3.51±0.44*^#^ |
| TC（mmol/L） | 3.07±0.14 | 6.54±0.05* | 5.05±0.06*^#^ |
| HDL（mmol/L） | 1.92±0.06 | 1.09±0.04* | 1.83±0.05*^#^ |
| ALT（U/L） | 42.56±4.04 | 49.06±10.76 | 46.24±3.50 |
| AST（U/L） | 159.60±11.54 | 171.07±15.31 | 170.30±12.07 |

NC, non-diabetic group as normal control; DM, non-treated diabetic group; DM+vild, diabetic mice with vild administration; FBG, fasting blood sugar; TG, triglyceride; HDL, high-density lipoprotein; TC, total cholesterol; ALT, alanine transaminase; AST, aspartate transaminase. One-way ANOVA, n=5 per group. **P*<0.05 compared to the NC group, ^#^ *P*<0.05 compared to the DM group.
