# Supplementary material for: Vildagliptin Attenuates Myocardial Dysfunction and Restores Autophagy via miR-21/SPRY1/ERK in Diabetic Mice Heart
Source: Front Pharmacol. 2021 Mar 18;12:634365. doi: 10.3389/fphar.2021.634365 (PMC8013777; doi:10.3389/fphar.2021.634365)
Supplement: Supplementary file 2 [file table2.docx]

**Table S2. Physical and biochemical parameters of mice in NC (WT and miR-21^-/-^) group and DM (WT and miR-21^-/-^) group (mean ± SD).**

|  | NC | miR-21^-/-^NC | DM | miR-21^-/-^DM |
| --- | --- | --- | --- | --- |
| Body weight（g） | 33.12±6.68 | 30.00±3.08 | 34.82±1.28^#^ | 31.78±2.62 |
| FBG（mmol/L） | 5.68±0.33 | 4.90±0.42 | 18.47±3.22*^#^ | 8.75±0.60*^#&^ |
| TG（mmol/L） | 1.41±0.20 | 0.90±0.06 | 4.64±0.96*^#^ | 1.38±0.05^&^ |
| TC（mmol/L） | 3.07±0.14 | 2.17±0.11* | 6.54±0.05*^#^ | 2.66±0.75^&^ |
| HDL（mmol/L） | 1.92±0.06 | 1.52±0.07* | 1.09±0.04*^#^ | 1.27±0.07*^#&^ |
| ALT（U/L） | 42.56±15.95 | 42.60±6.95 | 49.06±4.82 | 48.02±1.79 |
| AST（U/L） | 164.02±14.96 | 148.82±13.21 | 171.07±15.31^#^ | 162.94±16.46 |

NC, non-diabetic group as normal control; miR-21^-/-^ NC, miR-21 knockout and non-diabetic group; DM, non-treated diabetic group; miR-21^-/-^ DM, miR-21 knockout and diabetic group; FBG, fasting blood sugar; TG, triglyceride; HDL, high-density lipoprotein; TC, total cholesterol; ALT, alanine transaminase; AST, aspartate transaminase. One-way ANOVA, n=5 per group. **P*<0.05 compared to the NC group, ^#^*P*<0.05 compared to the miR-21^-/-^ NC group; ^&^*P*<0.05 compared to the DM group.
