# Supplementary material for: Vildagliptin Attenuates Myocardial Dysfunction and Restores Autophagy via miR-21/SPRY1/ERK in Diabetic Mice Heart
Source: Front Pharmacol. 2021 Mar 18;12:634365. doi: 10.3389/fphar.2021.634365 (PMC8013777; doi:10.3389/fphar.2021.634365)
Supplement: Supplementary file 3 [file table3.docx]

**Table S3. Physical and biochemical parameters of mice in DM, DM+vild, DM+AAV9 NC+vild and DM+AAV9+vild group (mean ± SD).**

|  | DM | DM+vild | DM+ AAV9 NC+vild | DM+AAV9+vild |
| --- | --- | --- | --- | --- |
| Body weight（g） | 34.82±1.28 | 32.44±5.38 | 32.36±3.96 | 34.56±3.76 |
| FBG（mmol/L） | 18.47±3.22 | 9.13±2.15* | 10.83±2.68* | 11.2±2.95* |
| TG（mmol/L） | 4.64±0.96 | 3.51±0.44* | 3.64±0.63* | 3.76±0.24* |
| TC（mmol/L） | 6.54±0.05 | 5.05±0.06* | 5.29±0.53* | 5.48±0.74* |
| HDL（mmol/L） | 1.08±0.04 | 1.83±0.05* | 1.51±0.28* | 1.37±0.45^#^ |
| ALT（U/L） | 49.06±4.82 | 46.24±3.50 | 47.48±3.01 | 47.22±2.00 |
| AST（U/L） | 171.07±15.31 | 170.30±12.07 | 188.90±17.34 | 182.88±15.33 |

DM, non-treated diabetic group; DM+vild, diabetic mice with vild administration; DM+AAV9 NC+vild, diabetic mice injected with no-load AAV9 and vild; DM+AAV9+vild, diabetic mice injected with AAV9-miR-21 and vild. FBG, fasting blood sugar; TG, triglyceride; HDL, high-density lipoprotein; TC, total cholesterol; ALT, alanine transaminase; AST, aspartate transaminase. One-way ANOVA, n=5 per group. **P*<0.05 compared to the DM group, ^#^*P*<0.05 compared to the DM+vild group; ^&^*P*<0.05 compared to the DM+AAV9 NC+vild group.
