# Supplementary material for: Vildagliptin Attenuates Myocardial Dysfunction and Restores Autophagy via miR-21/SPRY1/ERK in Diabetic Mice Heart
Source: Front Pharmacol. 2021 Mar 18;12:634365. doi: 10.3389/fphar.2021.634365 (PMC8013777; doi:10.3389/fphar.2021.634365)
Supplement: Supplementary file 4 [file table4.docx]

**Table S4. Primers sequenced for PCR.**

|  |  | Primer sequences (5’-3’) |
| --- | --- | --- |
| rno U6 | FORWARD | AGAGAAGATTAGCATGGCCCCTG |
|  | REVERSE | AGTGCAGGGTCCGAGGTATT |
|  | RT Primer | GTCGTATCCAGTGCAGGGTCCGAGGTATTCGCACTGGATACGACAAAAAT |
| mmu U6 | FORWARD | TGCGGGTGCTCGCTTCGGCAGC |
|  | REVERSE | CCAGTGCAGGGTCCGAGGT |
|  | RT Primer | GTCGTATCCAGTGCAGGGTCCGAGG |
| rno miR-21 | FORWARD | cgcgcgcgTAGCTTATCAGATGA |
|  | REVERSE | ATCCAGTGCAGGGTCCGAGG |
|  | RT Primer | GTCGTATCCAGTGCAGGGTCCGAGGTATTCGCACTGGATACGACTCAACA |
| mmu miR-21 | FORWARD | GCGCGTAGCTTATCAGACTGA |
|  | REVERSE | AGTGCAGGGTCCGAGGTATT |
|  | RT Primer | GTCGTATCCAGTGCAGGGTCCGAGGTATTCGCACTGGATACGACTCAACA |
