# Supplementary figures and images for: Vildagliptin Attenuates Myocardial Dysfunction and Restores Autophagy via miR-21/SPRY1/ERK in Diabetic Mice Heart
Source: Front Pharmacol. 2021 Mar 18;12:634365. doi: 10.3389/fphar.2021.634365 (PMC8013777; doi:10.3389/fphar.2021.634365)

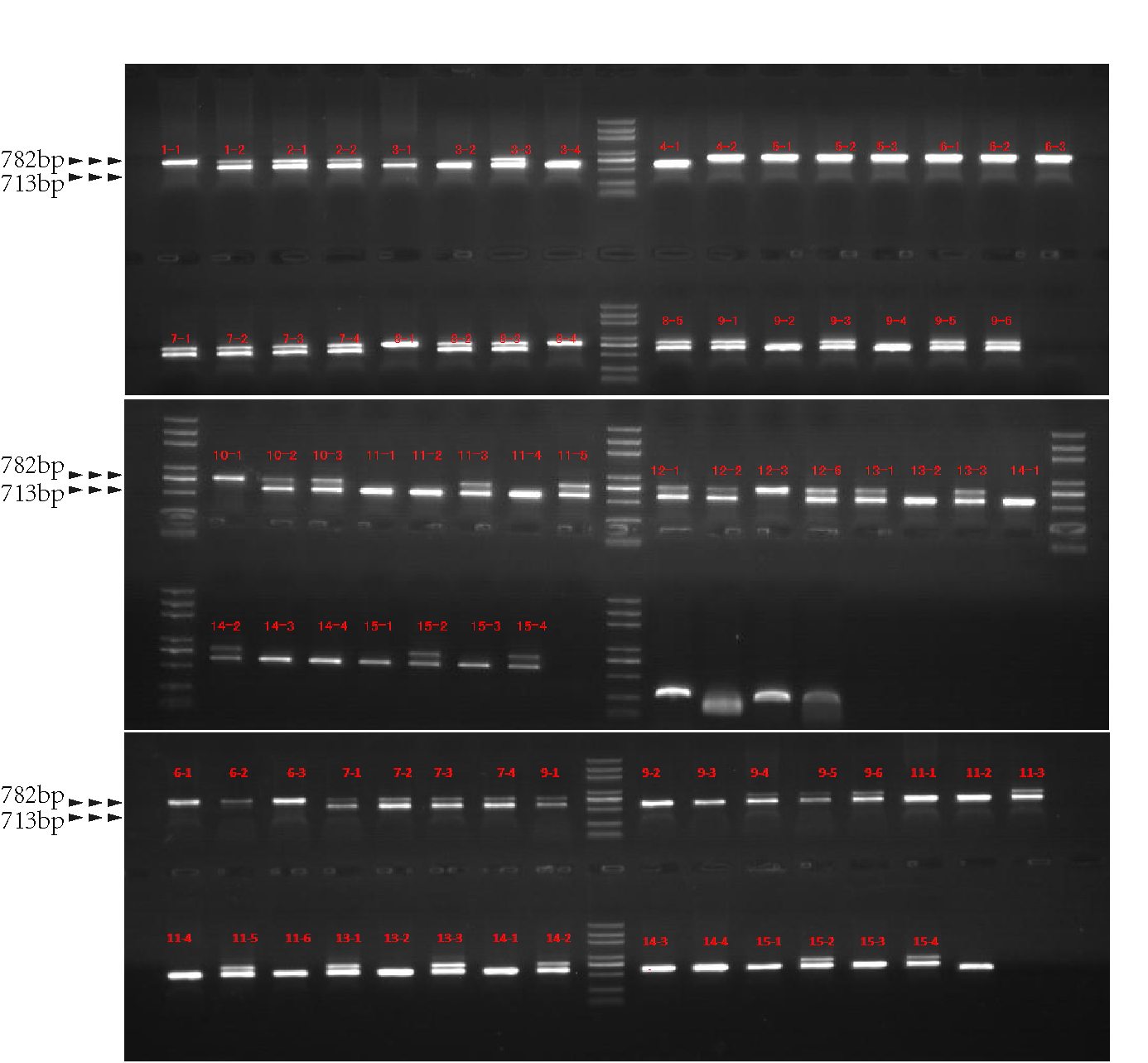

Supplement: Supplementary file 5 [file image1.tif]

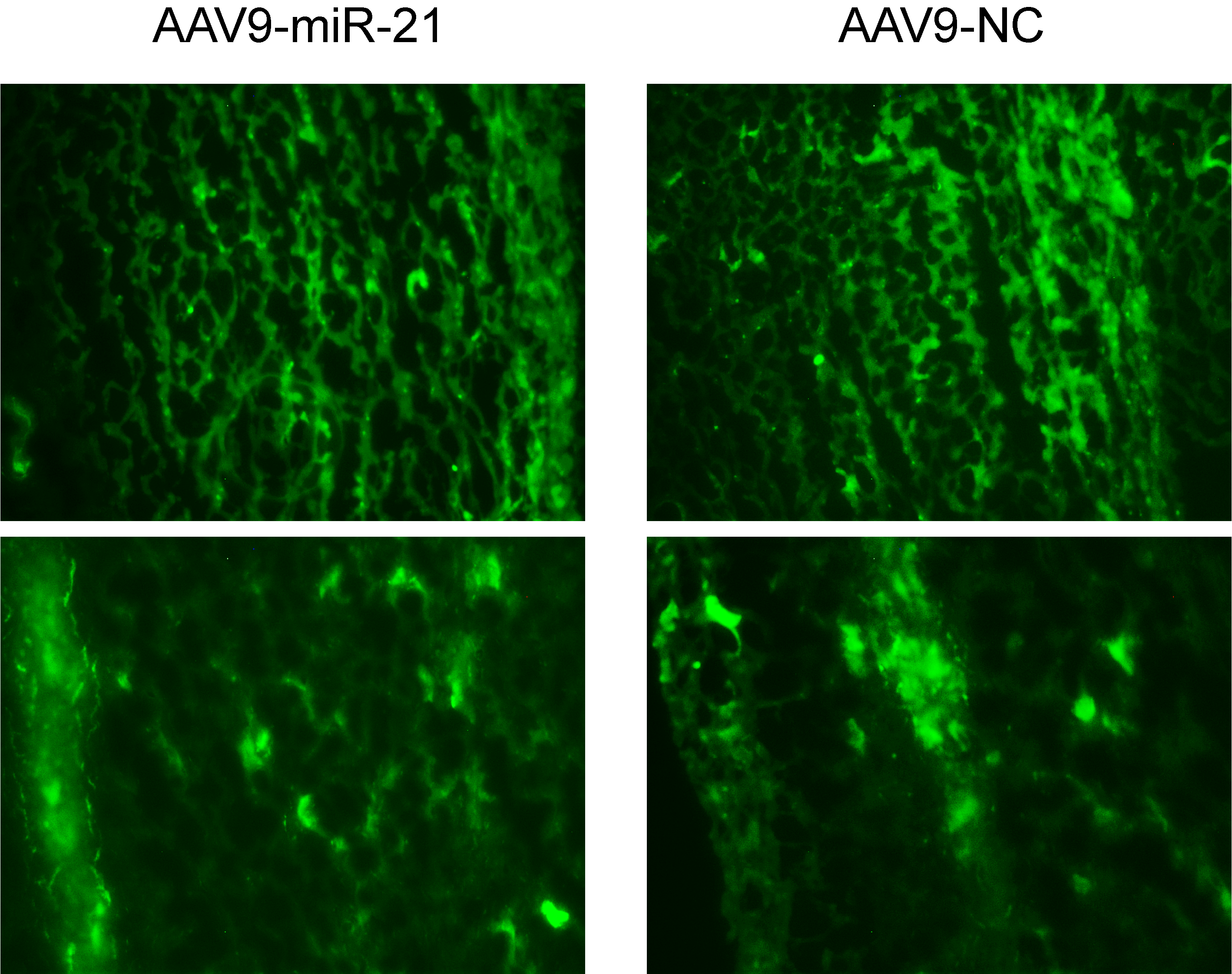

Supplement: Supplementary file 6 [file image2.tif]

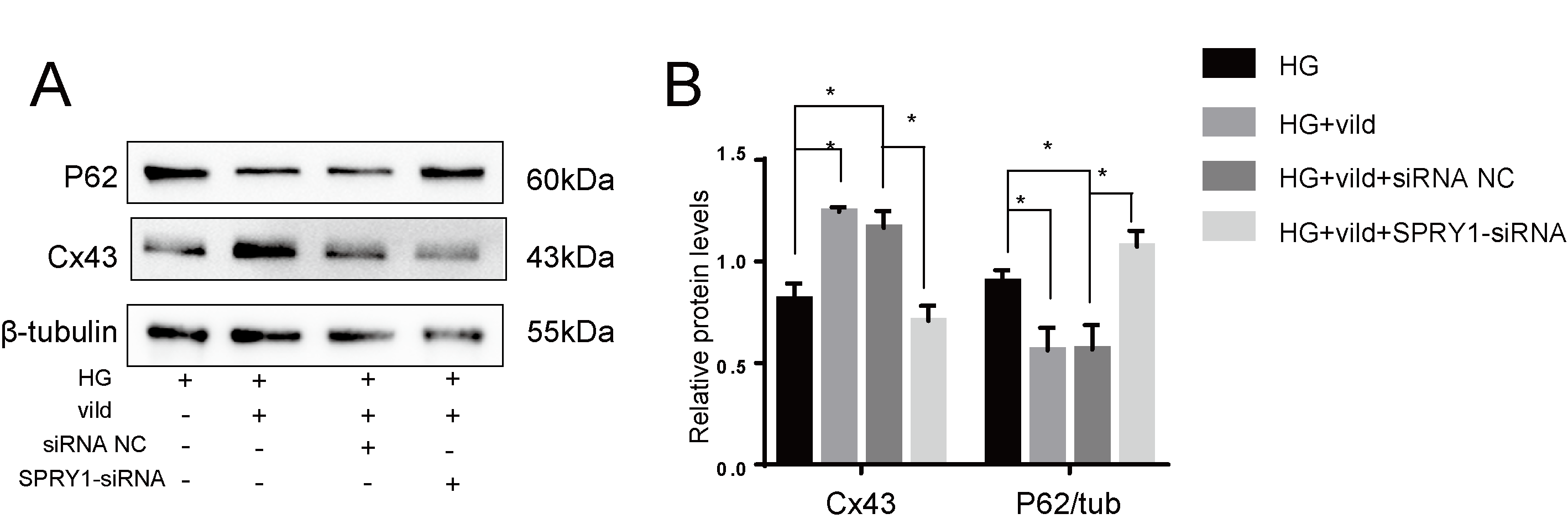

Supplement: Supplementary file 7 [file image3.tif]

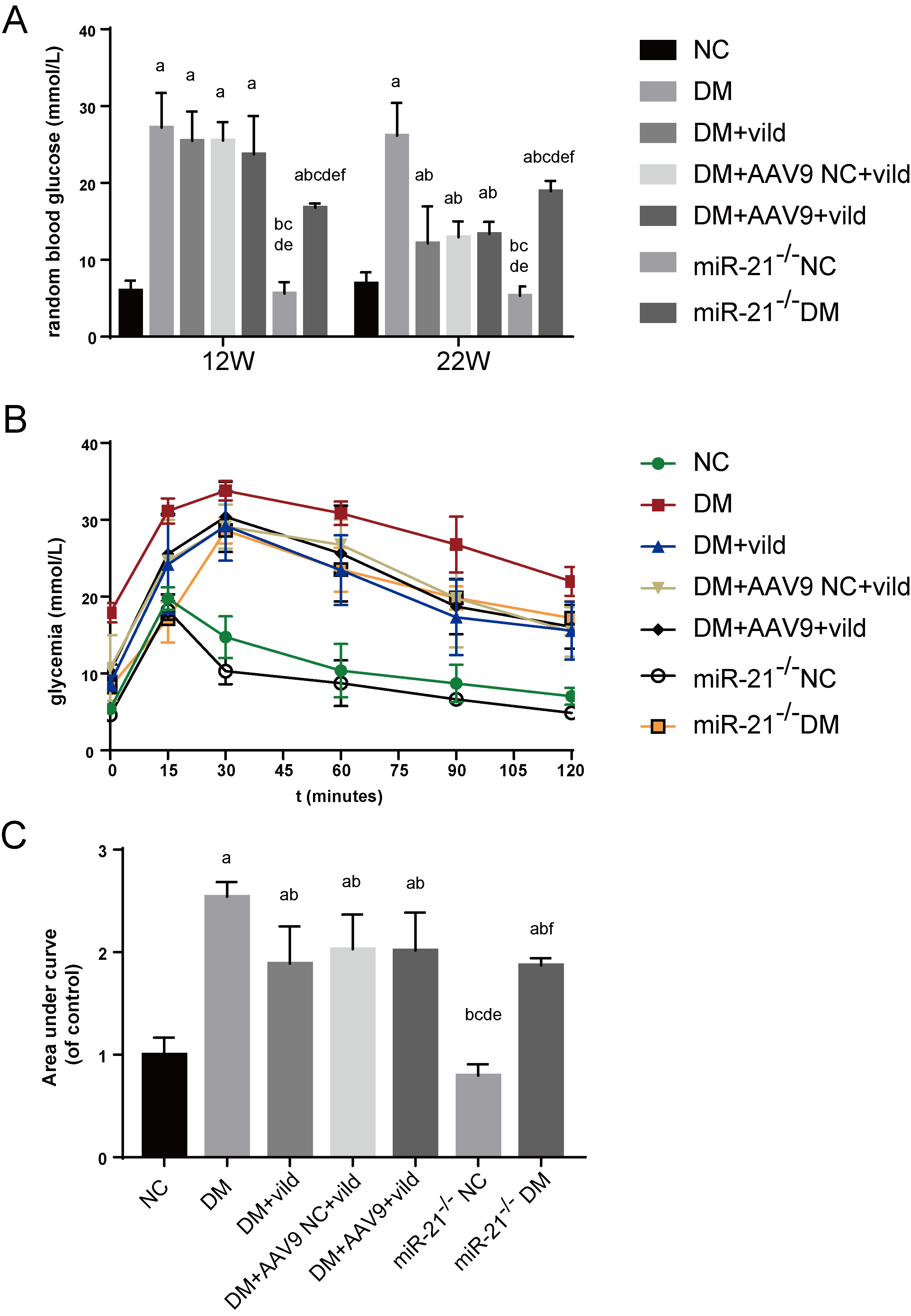

Supplement: Supplementary file 8 [file image4.tif]
